# Intragastric balloon insertion and pancreatitis: Case series

**DOI:** 10.1016/j.ijscr.2020.08.043

**Published:** 2020-08-29

**Authors:** Omar Alqabandi, Yousef Almutawa, Dana AlTarrah, Mufarrej Alhajeri, Mohammad H. Jamal, Sulaiman Almazeedi

**Affiliations:** a Amiri Hospital, Kuwait; b Jaber Al-Ahmad Hospital, Kuwait; c Faculty of Public Health, Kuwait University, Kuwait

**Keywords:** Pancreatitis, Intragastric balloon, Balloon pancreatitis, Obesity surgery

## Abstract

**Introduction:**

Obesity is a complex multifactorial disease that affects populations worldwide. In Kuwait, the prevalence of obesity is a major public health problem. Intra-Gastric Balloon (IGB) is commonly used as a non-operative strategy among bariatric patients. However, with the increasing use of IGB, life-threatening adverse outcomes are widely reported.

**Presentation of cases:**

A case series five patients presenting with pancreatitis from IGB in Kuwait is reported to better investigate the emerging complications of IGB. Three types of IGB were inserted, these include the Orbera Intragastric Balloon System, Orbera365 Intragastric Balloon System, and the Spatz Adjustable Gastric. The clinical course of balloon pancreatitis is described.

**Discussion:**

The case series examined existing case reports of IGB associated pancreatitis in the literature, in addition to the investigated clinical outcomes. Patients presented with mild pancreatitis, and removal of balloon resulted in significant improvement in symptoms. Pathogenesis of balloon pancreatitis could be secondary to the pancreas compression by the IGB.

**Conclusion:**

Despite the rarity of balloon pancreatitis, it needs to be recognized as a complication of IGB insertion. Further research is needed to better understand the implications of the balloon size, shape, volume and location of balloon insertion in order to prevent this fatal complication.

## Highlights

• Intra-Gastric Balloon use as non-operative strategy for bariatric patients.
• Rise of adverse life-threatening outcomes of Intra-Gastric Balloon.
• Pancreatitis could be secondary to pancreas compression by the Intra-Gastric Balloon.
• Need for the recognition of balloon pancreatitis as a complication of Intra-Gastric Balloon insertion.

## 1 Introduction

Obesity is recognized as major public health problem and has reached epidemic proportions worldwide [1]. In the State of Kuwait, the prevalence of obesity is alarmingly high, affecting 40% of adults [2]. The rising prevalence of obesity and its associated health related comorbidities highlight the need for effective treatment strategies to manage and reduce the burden of disease [3]. Bariatric surgery is considered the most effective treatment for morbid obesity, and is found to positively ameliorate related comorbidities, compared to conventional strategies [4]. However, although, bariatric surgery is primarily restricted to morbidly obese patients that meet a preoperative criteria (BMI > 35 with comorbidities, or BMI > 40), an intermediate group of patients that are not bariatric candidates that are also found to not respond well to medical therapy may benefit from surgery [5,6].

To address this intermediary group, in 1985 the Garren–Edwards Gastric Bubble (GEGB®) was introduced in the United States [7]. Initially, however, it was recommended that the intra-gastric balloons (IGB) are utilized only in clinical trials due to safety issues [8]. However, since then, several IGB were developed to meet pre-set standards for safety [9]. The uses of IGB vary, ranging from primary weight loss, weight loss solutions in high risk patients and as a bridge to bariatric surgery [10]. Table 1 summarizes the most popular types in used.

**Table 1:** Types of routinely used Intra-Gastric Balloons.

Balloon	Insertion method	Method of extraction	Volume	Estimated excess weight loss	Duration of implantation
Orbera [17]	Endoscopy	Endoscopy	450–750 ml liquid-filled	25%	6 months (Orbera 365–12 months)
Spatz adjustable balloon system [18]	Endoscopy	Endoscopy	400–800 ml liquid-filled	48%	12 months
Reshape Duo integrated Dualballoon system [19]	Endoscopy	Endoscopy	450 ml for each balloon, total 900 ml liquid-filled	25%	6 months
Elipse [20]	Patient swallows	Natural expulsion	550 ml liquid-filled	10%	16 weeks
Air supplied Obalon Gastric balloon [21]	Patient swallows	Endoscopy	Air-filled, 250 ml for each balloon (up to three balloons total 750 ml)	25%	6 months
Heliosphere BAH [22]	Endoscopy	Endoscopy	600 ml Air-filled	24%	6 months

The safety of IGB has been evaluated in multiple studies and is considered generally safe [11,12]. However, several life-threatening complications have been reported including gastric perforation, intestinal obstruction secondary to migration, and rarely acute pancreatitis [[13], [14], [15]]. In this case series, we shed light on the latter complication and present five patients who developed acute pancreatitis secondary to IGB insertion. Our aim is to investigate this emerging complication and propose that it is better recognized and listed as a possible post-procedure adverse event. The present case series is compliant with the PROCESS guidelines for case series [16].

## 2 Case series

### 2.1 Cases overview

Five cases of patients are described; three female and two males with a mean age of 23.6 years (SD ± 6.17), and a mean BMI of 34 kg/m^2^ (SD ± 6.17). As presented in Table 2 there were no critical past medical histories and no other apparent risk factors for pancreatitis. One patient (20%) was morbidly obese and four (80%) were actively practicing lifestyle modification. All patients consented prior to inclusion in this case series.

**Table 2:** Patient Background Information.

Patient	Age (years)	Gender	Weight (kg)	Height (cm)	BMI (kg/m^2^)	Past medical history	Past surgical history	Balloon type	Volume	Date of insertion	Place of insertion
Patient 1	23	Male	120	177	38.3	DLP, psoriasis, anxiety	Mini-abdominoplasty - 2014	Orbera	590 ml	21 Jan 2019	Kuwait
Patient 2	28	Female	75	170	25.9	None	Bilateral breast implants - 2016	Orbera	600 ml	15 Dec 2015	Kuwait
Patient 3	18	Female	105	160	41.0	None	None	Orbera	550 ml	August 2014	Kuwait
Patient 4	30	Male	110	177	35.1	None	None	Spatz	550 ml	May 2018	UAE
Patient 5	19	Female	77	161	29.7	None	None	Orbera	500 ml	30 Dec 2019	Kuwait

### 2.2 Intragastric balloon (IGB) insertion

In this case series, three types of IGB were inserted. These include the Orbera Intragastric Balloon System (Orbera, Apollo Endosurgery, Austin, Texas, USA), Orbera365 Intragastric Balloon System (Orbera, Apollo Endosurgery, Austin, Texas, USA), and the Spatz Adjustable Gastric Balloon (Spatz Medical, Great Neck, New York, USA).

All IGB were inserted by General Surgeons across several centers in Kuwait with an exception of the Spatz balloon which was inserted in the United Arab Emirates. There were no reported occurrences of intra-operative complications. The average volume injected into the IGB was 558 ml (range: 500–600, SD ± 39.6) of methylene blue with normal saline.

### 2.3 Post IGB complications

All the cases presented to clinical care from the emergency department across two centers in Kuwait. The clinical course of *balloon pancreatitis* is detailed in Table 3.

**Table 3:** Clinical Course of Balloon Pancreatitis.

Patient	Date of Diagnosis	Presentation	Duration of symptoms (days)	Labs	Imaging	Treatment	Balloon removed	Duration of treatment
Patient 1	23 October 2019	epigastric pain, nausea, vomiting	1	amylase 221 lipase 159 wbc 8.7 rft + lft normal	NA	npo, iv fluids, antiemetic, analgesic, proton pump inhibitor	No	1 day
Patient 2	13 January 2016	severe epigastric pain radiating to back, nausea	1	amylase 506, lipase 1214.8	NA	npo, iv fluids, antiemetic, analgesic, proton pump inhibitor	Yes	2 days
Patient 3	15 October 2014	constipation & no flatus, epigastric pain, nausea, repeated vomiting	2	amylase 96 urinary amylase 463 (high) lipase 135 wbc 10.3 rft + lft normal	CT: focal pancreatitis	npo, iv fluids, antiemetic, analgesic, proton pump inhibitor	Yes	5 days
Patient 4	27 May 2019	epigastric pain	1	amylase 665 lipase 323	Ultrasound normal	npo, iv fluids, antiemetics, analgesic	Yes	1 day
Patient 5	31 December 2019	nausea vomiting, epigastric pain	1	amylase 254 lipase 312.8	Ultrasound normal	npo, iv fluids, antiemetics, analgesic	No	1 day

On biochemical investigation, the mean levels of serum amylase and lipase were 422 U/L (SD ± 185) and 429 U/L (SD ± 448), respectively. Elevated amylase and lipase were observed in 100% and 60% of cases, respectively. All five cases were diagnosed and treated for acute pancreatitis. The mean duration from IGB insertion to the development of acute pancreatitis was 154 days (SD ± 170).

### 2.4 Management

The average length of stay under clinical care was 2 days. Two cases responded to conservative medical treatment while three cases required additional medical treatment and IGB removal. All five cases were kept nil per os (NPO) and received intravenous fluids, antiemetics, and analgesia. Three cases additionally received intravenous proton pump inhibitors. The chemical and biochemical details of cases are illustrated in Table 3.

### 2.5 Statistical analysis

To investigate whether factors can significantly influence the management outcome (IGB removal versus conservative medical treatment) in the management of *balloon pancreatitis*. Statistical analysis was carried out using SPSS (IBM SPSS statistics version 20). Descriptive statistics were carried out, mean and standard deviation was calculated for continuous variables, and frequencies and numbers for categorical variables.

The case series is registered with research registry, unique identifier number: researchregistry5842.

## 3 Discussion

The benefits of IGB in weight reduction are well documented in the literature. However, the present case series demonstrates that although rare, *balloon pancreatitis* is a significant complication among patients undergoing IGB insertion. A diagnostic triad was developed as a useful aid to diagnose the condition, and aid clinicians in recognizing the complication. The diagnostic criterion included: 1) recent gastric balloon insertion; 2) symptoms consistent with pancreatitis; 3) biochemical or radiological evidence of pancreatitis.

The removal of the balloon provided significant symptomatic relief in most patients, whereas two patients were managed conservatively without removing the IGB. Similar to previous case reports, patients in the present case series were found to present with mild pancreatitis [24,25,35]. Nevertheless, since severe course of pancreatitis has been described in the literature, it should be cautioned that not all *balloon pancreatitis* remain mild [23].

As summarized in Table 4, *balloon pancreatitis* remains to be a disease limited to case reports observed in centers that use IGB to aid weight reduction among bariatric patients. Although it has been suggested that an abdominal ultrasound should be performed to rule out the presence of gallstones, in most reports, the IGB is frequently removed. As such, in one case study, the presence of a distended gallbladder confounded the diagnosis of pancreatitis, which was believed to be secondary to an acute cholecystitis [34]. Despite performing a laparoscopic cholecystectomy, the patient did not improve symptomatically and eventually had the IGB removed. In another case series Alsohaibani et al. (2019), reported that of the 10 patients with *balloon pancreatitis,* five were treated conservatively [40]. Moreover, patients were found to present with a mild course of pancreatitis, and the greatest severity reported as a Bedside Index for Severity in Acute Pancreatitis (BISAP) score was 2 [40]. The authors also reported that patients who developed this condition was found to be following Heliosphere air-filled balloon insertion [40]. However, due to the limited number of reports on balloon *pancreatitis* due to an air-filled balloon system, it could be suggested that the underlying cause was related to direct physical pressure.

**Table 4:** Summary of case report using IGB to aid weight reduction in bariatric patients.

Study group	Number of patients	Type of balloon	Presentation after insertion	Method of diagnosis	Severity of Pancreatitis	Management
Abulrahman et al. [25]	1	BIB system	10 weeks	Lab, ultrasound, CT abdomen	Mild	Endoscopic removal
Issa et al. [24]	1	BIB	3 days	Lab, CT	Mild ^┼^	Endoscopic removal
Vongsuvanh et al. [23]	1	Spatz	11 months (3 months after adjustment)	Lab, CT	Severe	Exploratory laparotomy, removal of balloon
Torres et al. [33]	1	N/A	4 months	Lab, ultrasound, CT	N/A	Endoscopic removal
Öztürk et al. [34]	1	Spatz	1 month	Lab, ultrasound, CT, abdominal x-ray	N/A	Laparoscopic cholecystectomy, endoscopy, laparotomy
Shelton et al. [35]	1	Orbera	10 weeks	Lab, ultrasound, MRCP, CT	Mild	Endoscopic removal
Geffrier et al. [36]	1	Orbera	15 days	Lab, CT	N/A (Balthazar C)	Endoscopic removal
Gore et al. [37]	1	Orbera	1 day	Lab, ultrasound CT	N/A	Endoscopic removal
Said et al. [38]	1	N/A	5 weeks	Lab, ultrasound CT	N/A	Endoscopic removal
Aljiffry et al. [39]	1	N/A	4 months	Lab, ultrasound, MRCP, CT	N/A	Endoscopic removal
Alsohaibani [40]	10	Allergan, Medsil, Obalon, Orbera, Spatz, Heliosphere	1–494 days	Lab, ultrasound CT (done in 5 patients)	All ten patients had mild pancreatitis using the BISAP score^┼^	5 conservative, 5 endoscopic removal

These findings led to the proposition that the pathogenesis for *balloon pancreatitis* is secondary to the compression of the pancreas by the IGB, as observed during computed tomography (CT) imaging of the abdomen [25]. This aforementioned compression is likely to affect both the stomach and the pancreas. Compression of blood flow to the area of the stomach may even lead to localized ischemia resulting in ulcer formation and eventually perforation [[29], [30], [31]]. Giardello et al. suggested that the pathology is more obvious among patients with previous gastric surgery due to the alteration of the abdominal anatomy and blood supply to the stomach. Thus, some investigators recommend that previous gastric surgeries are an absolute contraindication for the insertion of IGB [32].

**Fig. 1:**
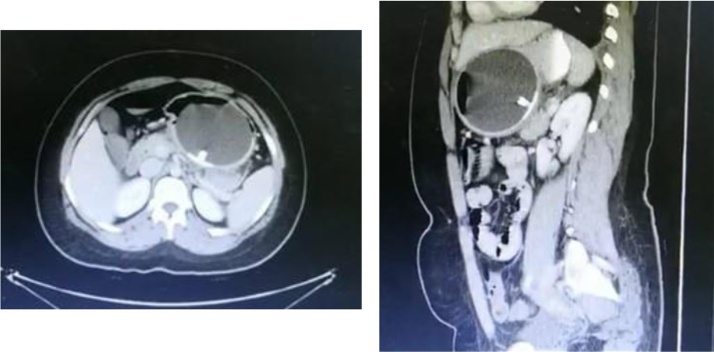
Computed tomography scan demonstrating compression of the pancreas by the intragastric balloon, obtained in case 1.

In this series, patients were considered healthy and did not undergo gastric surgery prior to the insertion of IGB. In Patient 1, imaging obtained demonstrated IGB compressing an acutely inflamed pancreas (Fig. 1). This highlights the accuracy of using CT scans to diagnose and stratify the severity of the condition in cases where no improvement is observed.

Several studies have shown the safety and efficacy profile for the use of IGB in bariatric patients [[26], [27], [28]]. These studies vary with regards to sample size, therefore may not detect the incidence of balloon pancreatitis. Although severe complications resulting from IGB have been reported [26], the incidence of *balloon pancreatitis* remains to be underreported. As more IGB are utilized in the management of bariatric patients globally, it is likely that the incidence of *balloon pancreatitis* will increase.

## 4 Limitations

Limitations of the case series include the small sample size which may have limited statistical analysis, and thus the standard error value could not be added to the mean value, as such only descriptive analysis was carried out. In addition to the small sample size, the retrospective nature of this paper limits the scope of our findings to a descriptive analysis, which cannot confer causation.

## 5 Conclusion

As previously discussed, the proposed pathogenesis for balloon pancreatitis may possibly be due to the compression of the pancreas by the IGB. Following the review of the literature, and clinical experience of the authors, the case series demonstrated that there appears to be a bias towards endoscopic removal of IGB. Nevertheless, in line with recommendations of other case reports, some patients may benefit from a more conservative approach. Given the rarity of the condition, it may be difficult to perform a randomized control trial in order to establish an evidence-based standard of care. Further research is needed to better understand the implications of the balloon shape, size, volume and location of insertion, in order to potentially prevent this fatal complication. Authors of the case series suggest that *balloon pancreatitis* is recognized as a complication of IGB insertion and that both patients and treating physicians are aware of this during the course of follow-up.

## Declaration of Competing Interest

No conflict of interest.

## Sources of funding

None.

## Ethical approval

Retrospective case series are exempt from ethical approval by the Ethical Committee at the Ministry of Health Kuwait.

## Consent

Written informed consent was obtained from the publication of the case series.

## Author contribution

All authors contributed equally to the data collection, analysis and write-up of this case series.

## Registration of research studies

The case series is registered with research registry, unique identifier number: researchregistry5842.

## Guarantor

Sulaiman Almazeedi.

## Provenance and peer review

Not commissioned, externally peer-reviewed.

## CRediT authorship contribution statement

**Omar Alqabandi:** Investigation, Writing - review & editing. **Yousef Almutawa:** Investigation, Writing - review & editing. **Dana AlTarrah:** Writing - review & editing. **Mufarrej Alhajeri:** Investigation, Writing - review & editing. **Mohammad H. Jamal:** Investigation, Writing - review & editing. **Sulaiman Almazeedi:** Conceptualization, Writing - review & editing.
