# Supplementary material for: Intragastric balloon insertion and pancreatitis: Case series
Source: Int J Surg Case Rep. 2020 Aug 29;74:263–7. doi: 10.1016/j.ijscr.2020.08.043 (PMC7486575; doi:10.1016/j.ijscr.2020.08.043)
Supplement: Supplementary file 1 [file mmc1.doc]

**Supplementary Table 1: Patient Details (1 out 2)**

| **Variable** | **Total**  **(N=5)** | **Balloon Removed (N=3)** | **Conservative (N=2)** |
| --- | --- | --- | --- |
| Age (years), mean ± SD | 23.6 ± 5.32 | 25.3 ± 6.43 | 21.0 ± 2.83 |
| Weight (kg), mean ± SD | 97.4 ± 20.3 | 96.7 ± 18.9 | 98.5 ± 30.4 |
| Height (cm), mean ± SD | 169 ± 8.28 | 169 ± 8.54 | 169 ± 11.3 |
| Body mass index (kg/m^2^), mean ± SD | 34.0 ± 6.17 | 34.0 ± 7.59 | 34.0 ± 6.08 |
| Morbid obesity, % | 20 | 33 | 0 |
| Lifestyle modification (yes), n | 4 | 3 | 1 |
| Lifestyle modification (no), n | 1 | 0 | 1 |
| Surgical history (yes), n | 2 | 1 | 1 |
| Surgical history (mini-abdominoplasty) (yes), n | 1 | 0 | 1 |
| Surgical history (bilateral breast implants) (yes), n | 1 | 1 | 0 |
| Comorbidities (all), n | 1 | 0 | 1 |
| Comorbidities (dyslipidemia), n | 1 | 0 | 1 |
| Comorbidities (psoriasis), n | 1 | 0 | 1 |
| Comorbidities (anxiety), n | 1 | 0 | 1 |
| Comorbidities (none), n | 4 | 3 | 1 |
| Used Orbera gastric balloon, n | 3 | 2 | 1 |
| Used Orbera 365 gastric balloon, n | 1 | 0 | 1 |
| Used Spatz gastric balloon, n | 1 | 1 | 0 |
| Gastric Balloon Volume (ml), mean ± SD | 558 ± 39.6 | 567 ± 28.9 | 545 ± 63.6 |
| Duration of Balloon stay (days), mean ± SD | 154 ± 170 | 165 ± 198 | 138 ± 192 |
| Signs & symptoms (yes), n | 5 | 3 | 2 |
| 1. Epigastric pain), n | 4 | 2 | 2 |
| 1. Severe epigastric pain, n | 1 | 1 | 0 |
| 1. Nausea), n | 4 | 2 | 2 |
| 1. Vomiting), n | 2 | 0 | 2 |
| 1. Constipation & no flatus), n | 1 | 1 | 0 |
| Examination (abdomen soft & lax), n | 5 | 3 | 2 |

**Supplementary Table 1: Patient Details (2 out 2)**

| **Variable** | **Total**  **(N=5)** | **Balloon Removed (N=3)** | **Conservative (N=2)** |
| --- | --- | --- | --- |
| NPO, IV fluids, antiemetic, analgesic, proton pump inhibitor (n), n | 3 | 2 | 1 |
| NPO, IV fluids, antiemetics, analgesic, n | 2 | 1 | 1 |
| Treatment duration (days), mean ± SD | 2 ± 2 | 3 ± 2 | 1 ± 0 |
| Imaging (normal), n | 2 | 1 | 1 |
| Imaging (pancreatitis), n | 1 | 1 | 0 |
| Imaging (NA), n | 2 | 1 | 1 |
| Amylase (U/L), mean ± SD | 422 ± 185 | 545 ± 106 | 238 ± 23.3 |
| Lipase (U/L), mean ± SD | 429 ± 448 | 558 ± 577 | 236 ± 109 |
| Amylase-normal, n | 0 | 0 | 0 |
| Amylase-pancreatitis suspected (> 200 U/L), n | 5 | 3 | 2 |
| Lipase-normal, n | 2 | 1 | 1 |
| Lipase-pancreatitis suspected (> 200 U/L), n | 3 | 2 | 1 |
| Amylase-normal, % | 0 | 0 | 0 |
| Amylase-pancreatitis suspected (> 200 U/L), % | 100 | 100 | 100 |
| Lipase-normal, % | 40 | 33 | 50 |
| Lipase-pancreatitis suspected (> 200 U/L), % | 60 | 67 | 50 |
| No gallbladder stones on USG, n | 5 | 3 | 2 |
